# Changes in lipids over twelve months after initiating protease inhibitor therapy among persons treated for HIV/AIDS

**DOI:** 10.1186/1476-511X-4-4

**Published:** 2005-02-10

**Authors:** Adrian R Levy, Lawrence McCandless, P Richard Harrigan, Robert S Hogg, Greg Bondy, Uchenna H Iloeje, Jayanti Mukherjee, Julio S Montaner

**Affiliations:** 1 Department of Health Care and Epidemiology, University of British Columbia (UBC), Vancouver, Canada; 2 Centre for Health Evaluation & Outcome Sciences, St. Paul's Hospital, Vancouver, Canada; 3 BC Centre for Excellence in HIV/AIDS, St. Paul's Hospital, Vancouver, Canada; 4 St. Paul's Hospital HIV Metabolic and Lipid Clinic, Vancouver, Canada; 5 Bristol-Myers Squibb, Wallingford, Connecticut, USA; 6 Department of Medicine, UBC, Vancouver, Canada

**Keywords:** HIV/AIDS, protease inhibitors, cholesterol, triglycerides, cardiovascular disease

## Abstract

**Background:**

Protease inhibitors are known to alter the lipid profiles in subjects treated for HIV/AIDS. However, the magnitude of this effect on plasma lipoproteins and lipids has not been adequately quantified.

**Objective:**

To estimate the changes in plasma lipoproteins and triglycerides occurring within 12 months of initiating PI-based antiretroviral therapy among HIV/AIDS afflicted subjects.

**Methods:**

We included all antiretroviral naïve HIV-infected persons treated at St-Paul's Hospital, British Columbia, Canada, who initiated therapy with protease inhibitor antiretroviral (ARV) drugs between August 1996 and January 2002 and who had at least one plasma lipid measurement. Longitudinal associations between medication use and plasma lipids were estimated using mixed effects models that accounted for repeated measures on the same subjects and were adjusted for age, sex, time dependent CD4+ T-cell count, and time dependent cumulative use of non-nucleoside reverse transcriptase inhibitors and adherence. The cumulative number of prescriptions filled for PIs was considered time dependent. We estimated the changes in the 12 months following any initiation of a PI based regimen.

**Results:**

A total of 679 eligible subjects were dispensed nucleoside analogues and PI at the initiation of therapy. Over a median 47 months of follow-up (interquartile range (IQR): 29–62), subjects had a median of 3 (IQR: 1–6) blood lipid measurements. Twelve months after treatment initiation of PI use, there was an estimated 20% (95% confidence interval: 17% – 24%) increase in total cholesterol and 22% (12% – 33%) increase in triglycerides.

**Conclusions:**

Twelve months after treatment initiation with PIs, statistically significant increases in total cholesterol and triglycerides levels were observed in HIV-infected patients under conditions of standard treatment. Our results contribute to the growing body of evidence implicating PIs in the development of blood lipid abnormalities. In conjunction with the predominance or men, high rates of smoking, and aging of the treated HIV-positive population, elevated lipoproteins and triglycerides may mean that patients such as these are at elevated risk for cardiovascular events in the future.

## Introduction

Abnormalities in the lipid metabolism of persons infected with human immunodeficiency virus (HIV), potentially induced by the disease itself and the medications used for treatment, were first reported in the early 1990s[1]. Reductions in high- (HDL) and low-density lipoprotein (LDL) cholesterol were observed amongst persons infected with HIV and increases in triglycerides were observed among persons with AIDS[1]. Following the introduction of protease inhibitors (PI), morphological changes in fat distribution were reported. This was followed by numerous reports of metabolic disturbances, including glucose and lipid abnormalities presenting as insulin resistance, impaired glucose tolerance, hyperglycemia, type 2 diabetes mellitus [2-5], elevated serum triglycerides, LDL and very low density lipoprotein cholesterol, apolipoprotein B, E, and lipoprotein(a) [2-4,6]. The combination of metabolic disturbances and morphological changes are now described as the HIV-related "lipodystrophy syndrome"[2,3].

Up to one-half of subjects treated with PIs have shown elevated levels of triglycerides, total cholesterol (TChol), LDL, insulin, and fasting glucose [7-10]. The onset of metabolic changes appears to occur soon after initiation of treatment, often as quickly as within several weeks[11]. The high prevalence of disturbances of lipoproteins and triglycerides, the rapidity of their onset, and large changes that have been observed in randomized trials have led to concern regarding the potential impact of PIs on the cardiovascular health of persons with HIV/AIDS. However, the direction and magnitude of changes in plasma lipoproteins and triglycerides induced by PIs has yet to be quantified in an observational setting. The aim of this study was to quantify the magnitude of change in lipoprotein and triglyceride levels over twelve months following any initiation of PI-based treatment in a cohort of subjects treated for HIV/AIDS in a large tertiary care institution.

## Methods

We included all antiretroviral naïve HIV-infected persons treated at St-Paul's Hospital, British Columbia (BC), Canada, who initiated use of PIs between August 1996 and January 2002. Plasma lipoprotein and triglyceride levels were obtained from measurements taken over the course of regular monitoring. Longitudinal effects of the impact of PIs on lipoproteins and triglycerides were estimated using statistical models that accounted for correlation due to repeated measurements on the same individuals.

### Study Setting and Population

In BC, antiretroviral drugs have been centrally distributed at no cost to eligible HIV-infected individuals since 1986[12,13]. In October 1992, the HIV/AIDS Drug Treatment Program became the responsibility of the BC Centre for Excellence in HIV/AIDS. Since December 1996, the mainstay of treatment for HIV/AIDS has been highly active antiretroviral therapy (HAART) including two nucleosides and either a PI or a non-nucleoside reverse transcriptase inhibitor (NNRTI). Typically, HIV-infected subjects receiving antiretroviral therapy are monitored by physicians at intervals no longer than three months at which time prescriptions are renewed or modified based on clinical and laboratory parameters, and necessary laboratory tests are conducted. This research received ethical approval from the Institutional Review Board of Providence Health Care in BC.

### Exposure to antiretroviral therapy for HIV/AIDS

At the BC Centre for Excellence in HIV/AIDS, records of CD4+ T-cell counts and a profile of dispensed antiretroviral therapy are routinely maintained, including the: prescription fill dates, medications prescribed, and amount dispensed. Records of dispensed antiretroviral medications, (including nucleoside analogs, PI and NNRTI) were used to determine the drugs dispensed and available to each subject during each month of follow-up. Subjects were considered unexposed until the first month that a PI was dispensed. The exposure at the time of the lipid measurement was calculated as the cumulative number of consecutive months of drug exposure up until and including the month in which a measurement was taken. As we were interested in estimating changes that occur in plasma lipoproteins and triglycerides within the first year after treatment initiation, we included measurements with a cumulative PI exposure up to a maximum of twelve months. Subjects were considered unexposed thirty days after the last PI was dispensed and, at that time, the cumulative exposure was set to zero.

### Plasma lipoproteins and triglycerides

Approximately one-half of HIV-infected patients in BC are followed at one treatment centre located at St. Paul's Hospital, a large teaching hospital in Vancouver. For these subjects, in addition to virological testing, the hospital laboratory records the results of plasma lipoproteins and triglycerides. Subjects were routinely instructed to fast for 12 hours prior to the sample being drawn. Blood samples were collected in 10-1 EDTA-coated vacutainer tubes. Plasma was separated by centrifugation for 10 minutes at 2,000 revolutions per minute. TChol in the plasma was determined using an enzymatic method[14] and plasma triglyceride was determined as previously described[15]. HDL cholesterol was determined using a heparin manganese precipitation of apo B-containing lipoproteins[16] and LDL cholesterol was calculated using the Friedewald formula[17,18].

For some subjects, the first lipid measurement was taken prior to the dispensation of any antiretroviral drug ("treatment naïve" baseline lipid measurements). Other subjects had their first recorded plasma lipoproteins and triglycerides after HAART was initiated (initiation lipid levels).

### Statistical analysis

We used linear mixed effects models[19] to estimate the effect of PI-based HAART on changes in plasma lipoproteins and triglycerides over twelve months. As blood lipid levels were always greater than 0, the responses were log transformed prior to model fitting. Effect estimates were obtained via restricted maximum likelihood estimation using the R^© ^function lme(.). An analysis of variance indicated that simple parallel-line models, where the exposure effect was assumed to be constant over subjects, could adequately explain variation in the data compared to more complex models with random slopes. To account for correlated responses due to repeated measurements on the same individual, the correlation structure of the model was specified to include only a random intercept for each subject.

The following explanatory variables were included in the models: age, sex, and CD4+ T- cell count at PI initiation, concomitant use of NNRTI, and a measure of adherence to antiretroviral therapy. Exposure to NNRTI was considered time-dependent and quantified using the same algorithm that was used to calculate time dependent PI exposure. Adherence was calculated by dividing the number of months of antiretroviral medications dispensed by the number of months of follow-up in the first twelve months after treatment initiation. Incomplete adherence represents the gap between the time that the previous medication supply ran out until the next refill date, and/or until the last contact date with the program. This method is reliably associated with both clinical outcomes and un-timed drug level monitoring[20,21].

For each outcome and associated regression coefficient β, the quantity exp(β)-1 represented the adjusted monthly percent change in that plasma lipid fraction. These values were annualized using the formula exp(12 × β)-1. Ninety five percent confidence intervals (95% CI) for the effect estimates were obtained using the standard error for each regression coefficient.

Two sensitivity analyses were conducted. The first analysis involved restricting the analysis to lipid measurements which were taken on subjects prior to any change from baseline antiretroviral therapy. The second sensitivity analysis included only subjects who had lipid measurements taken prior to initiation of therapy.

## Results

There were 679 subjects who were eligible for analysis, 91% of whom were male (Table 1). The median age was 38 years at initiation of therapy and the median baseline CD4+ T-CELL COUNT was 210 cells/mm^3^. All subjects initiated therapy that included a nucleoside analog and a PI.

**Table 1:** Demographic and clinical characteristics at treatment program enrollement among 679 subjects treated with PI-based HAART initiation for HIV/AIDS at St Paul's Hospital, Vancouver, BC, August 1996 – January 2002

Characteristic*	
Age (y)	
Mean (SD) Median	39 (8.9) 38
Sex (% male)	91
Status (% alive) at end of follow-up	93
CD4 (cells/mm^3^)	
Mean (SD) Median	255 (222) 210

Abbreviations: SD = Standard deviation; PI protease inhibitor.

Subjects were followed for a median of 47 months (Table 2). During that follow-up time, subjects had a mean of 73% of months in which a PI had been dispensed, with a mean of 31 prescription refills. The mean adherence in the first year was 89%, and the median adherence was 100% indicating that more than one-half of subjects were dispensed all their medications.

A total of 3,010 lipid measurements were used in the analysis, with 400 subjects having had two or more lipid measurements during the course of follow-up. There were a median of 3 (IQR 1–6) measurements per subject. The median time between lipid measurements was 3 (IQR 2–5) months for all subjects.

**Table 2:** Use of PIs among 679 subjects initiating PI-based HAART for HIV/AIDS at St Paul's Hospital, Vancouver, BC, August 1996 – January 2002

Characteristic*	
Number of months between initiation of PI and end of observation, median (IQR)**	47 (29–62)
% of follow-up time with dispensed therapy mean (SD), median for PI	73 (30) 84
Mean (SD) number prescriptions for***	
PI	31 (19)
% adherence in first year	
mean (SD)	89 (21)
median (IQR)	100 (92 – 100)

Abbreviations: SD = Standard deviation; IQR = Interquartile range; NRTI = nucleoside reverse transcriptase inhibitors; PI protease inhibitor; NNRTI = non-nucleoside reverse transcriptase inhibitor

* PIs available during follow-up included: indinavir, nelfinavir, saquinavir, and ritonavir. All data taken from time of first therapy initiation (treatment program enrollement).

** Time between first dispensation of HAART (enrollment) and last date of follow-up.

*** All prescriptions are of 30 day duration

Table 3 shows the percentage change in plasma lipoprotein and triglyceride levels after 12 months of PI use, after adjustment for age, sex, and CD4+ T- cell count at initiation, concomitant use of NNRTI, and adherence. Statistically significant increases of about 20% were observed in TChol, the lipoprotein fractions, and triglycerides.

**Table 3:** Baseline plasma cholesterol and triglycerides and estimated percent changes after 12 months when using PI among 679 subjects treated for HIV/AIDS at St Paul's Hospital, Vancouver, BC, August 1996 – January 2002

	**Number of measurements (subjects)**	**Mean lipid baseline measurements (SD)**	% change* (95% CI)
TChol	1620 (529)	4.2 (1.0)	20 (17, 24)
HDL	1250 (419)	1.0 (0.3)	22 (15, 29)
LDL	677 (295)	2.5 (0.8)	12 (5, 20)
N-HDL	1247 (419)	3.1 (1.2)	20 (15, 26)
TRG	1743 (556)	1.9 (1.2)	22 (12, 33)

Abbreviations: PI protease inhibitor; TChol = total cholesterol; HDL = high-density lipoprotein cholesterol; LDL = low- density lipoprotein cholesterol; N-HDL = Non-HDL Cholesterol; TRG = triglycerides

* Adjusted for age, sex, and CD4^+ ^cell count at first prescription for HAART, concomitant use of non-nucleoside reverse transcriptase inhibitor and adherence to antiretroviral therapy in the first year of treatment

The sensitivity analyses indicated that the models were robust to changes in the sub-population studied and the exposures that were included. In the analysis that included subjects who had a baseline measurement prior to start of HAART, a plasma profile prior to antiretroviral therapy initiation showed a similar pattern but with wider confidence intervals. In the analysis which was restricted to lipid measurements taken on subjects prior to switching from baseline therapy, the effect estimates were similar to those in Table 3.

## Discussion

In patients treated for HIV infection with HAART in a naturalistic setting, we observed that treatment with PIs was estimated to result in 20% increases in the levels of total cholesterol, HDL- and LDL-cholesterol and triglycerides twelve months after treatment initiation. Our results contribute to the growing body of evidence implicating PIs in the development of blood lipid abnormalities[22] and are in keeping with findings of randomized trials where a change from PI-based HAART to NRTI- or NNRTI-based HAART was associated with improvements in the lipid profile[23]. The observed increases in LDL-cholesterol and triglycerides were expected based on results reported from randomized trials. The finding of an increase in HDL-cholesterol has been reported only in some[24] but not all randomized trials.

Low HDL levels and other forms of dyslipidemia, and disturbances in glucose metabolism, as well as central obesity, have been shown to be strong independent risk factors for cardiovascular morbidity and mortality in the general population[25,26]. Furthermore, the clustering of risk factors leads to greater cardiovascular morbidity and mortality than would be expected to occur in relation to each component alone[25]. These factors, when considered together, provide grounds to suspect that persons being treated for HIV infection with PI are at an increased risk of cardiovascular disease[8]. The increases in triglycerides caused by use of PI is of concern because there is growing evidence that these lipids are an independent risk factor for cardiovascular disease[27,28].

Two large observational cohort studies, published in 2003, showed discrepant results regarding the risks of cardiovascular disease among persons treated with HAART[29,30]. A retrospective administrative claims database study from the US Veteran's Affairs indicated that there was no relation between the use of antiretroviral therapy and the risk of cardiovascular or cerebrovascular hospitalizations [29]. A prospective multi-country collaborative study, with clinical events validation from eleven cohorts of HIV-infected persons showed that the incidence of myocardial infarction increased with longer exposure to combination antiretroviral therapy[30].

This study has a number of features that add credence to the results. First, while only based at one hospital, the study population comprised about one-half of the treated population in BC. The study sample had similar demographic composition (age and sex) and CD4+ T-cell count at initiation of therapy as other treated subjects in the province. We believe that these results are therefore generalizable to PI-regimen treated HIV-infected patients in BC, as well as to other target populations with similar demographic characteristics. Second, as all dispensed PIs and other antiretroviral medications are paid for and recorded centrally, there was complete information on all HAART medications available to all study subjects. We adjusted for adherence using each subjects' refill compliance in the first year of treatment. Third, data on the plasma lipid profile, including both lipoproteins and triglycerides, were of high quality. The measurements, while lacking the feature of being taken at uniformly spaced intervals as in closely monitored system such as randomized trial, represent the actual experience in an observational setting. Fourth, the enrollment period lasted over five years, allowing a large sample size. We employed repeated measures analyses to account for the correlated structure of the data.

This study was also subject to several limitations. First, lipid measurements were not collected in a predetermined manner as is typical in a prospective research study. Consequently, the timing of measurements was irregular and not all lipid fractions were measured when blood was drawn. Second, despite instructions some subjects may not have fasted for the full 12 hour period prior to blood draws. Non-fasting triglyceride measurements are still useful for predicting cardiovascular disease and death[31]. Third, other potent risk factors for changes in lipid profiles such as family history and diet were not considered. It is possible but unlikely that the type of triple therapy prescribed by physicians could have been influenced by the belief that PIs may negatively impact lipid levels, likely leading to an attenuation of the estimates. Fourth, a number of factors contributed to a bias towards underestimating the true changes: for some subjects baseline values were not obtained prior to treatment initiation; it was not possible to adjust for concomitant use of lipid modifying agents such as statins because these data were not available; subjects with large changes in their lipid profiles may have been switched to another class; and treatment interruptions would also have affected exposure estimates. As a result of these limitations, the actual changes in plasma lipoprotein and triglycerides may be greater than reported in this study. Fifth, we assumed that patients who stop therapy or skip a month of therapy can be modelled as treatment naïve with respect to any subsequent therapy. More sophisticated statistical techniques are available[32] for handling this limitation, but appropriate software and resolution of technical issues are still in the developmental stages. Lastly, it is possible that the PI effect on lipids and triglyceride levels is not a class effect. The recently introduced PI, atazanavir sulfate, has been shown in several randomized clinical trials not to adversely affect lipid levels [33-38].

Due to changes in the HAART regimen that occurred because of resistance, non-compliance, or other reasons, it was not possible to isolate the effect of individual PIs. Over the period under study, most subjects initiating PI-based HAART were prescribed either indinavir or nelfinavir.

### Treating PI-induced dyslipidemia

As PI-based HAART is very effective in increasing survival in HIV-infected patients[39], discontinuing PI is undesirable, even in patients with dyslipidemia. However, as PI-induced dyslipidemia is often asymptomatic and typically occurs in younger patients whose baseline risk of cardiovascular disease is low, the need for primary prevention is often under-appreciated in clinical practice[40]. When it is recognized, rather than discontinuing or switching PI therapy, one response is to initiate pharmacologic therapy with statins and/or fibrates[41].

Statins have been shown to be effective drugs to prevent cardiovascular disease in non-HIV patients[26]. While the role of statins in preventing cardiovascular disease in HIV patients remains to be demonstrated definitively, several smaller randomized controlled trials that have shown that these medications have beneficial effects on PI-induced dyslipidemia [42]. Until recently, pravastatin was the preferred statin for treating PI-induced dyslipidemia because it does not require metabolism by the cyp 3A4 system. However, recent studies have shown that moderate lipid lowering with pravastatin was less effective than intensive lipid lowering with atorvastatin in reducing: progression of atherosclerotic lesions [43] in patients requiring coronary angiography; and death or major cardiovascular events in patients with an acute coronary syndrome[44]. Atorvastatin is a powerful and effective statin, though there are still only limited published studies with this agent in HIV patients[45]. Because of metabolism by cyp 3A4, the dose of atorvastatin should be reduced one-half in patients receiving PIs. Lovastatin and simvastatin require metabolic activation by cyp 3A4 and should be avoided in patients receiving PIs. Rosusvastatin, a recently introduced statin has features that make it attractive for treating patients with PI-induced dyslipidemias: it does not require metabolism by the cyp 3A4 system and it effectively reduces LDL cholesterol. The safety and effectiveness of rosuvastatin in reducing clinical outcomes in HIV patients remain to be demonstrated.

Fibrates are effective in lowering triglycerides and increasing HDL and have been shown to be effective in HIV patients[42]. Despite the impact on plasma lipids and triglycerides, only one trial has shown fibrates to reduce clinical outcomes[46,47]. Therefore, the role of fibrates in reducing cardiovascular disease outcomes remains to be fully elucidated. The main role of fibrates is to reduce the risk of pancreatitis associated with hypertriglyceridemia. In HIV patients, gemfibrozil should be limited to monotherapy because of the risk for myopathy. Fenofibrate is the preferred drug if combination therapy with a statin is contemplated. The efficacy of other lipid lowering drugs such as niacin and cholesterol transport blockers (ezetimibe) remains to be demonstrated. Bile acid sequestrants are contraindicated in patients receiving HAART.

We conclude that there was an estimated 20% increase in lipoproteins and triglycerides in the first year after initiating PI-based antiretroviral therapy in HIV-infected patients under conditions of standard treatment. Whether these increases continue beyond one year is of considerable interest because lipoproteins and triglycerides are known risk factors for cardiovascular disease. In conjunction with the predominance or men, high rates of smoking, and aging of the treated HIV-positive population, elevated lipoproteins and triglycerides may mean that patients such as these are at elevated risk for cardiovascular events in the future.

## References

[B1] Grunfeld C, Pang M, Doerrler W, Shigenaga JK, Jensen P, Feingold KR (1992). Lipids, lipoproteins, triglyceride clearance, and cytokines in human immunodeficiency virus infection and the acquired immunodeficiency syndrome. J Clin Endocrinol Metab.

[B2] Carr A, Samaras K, Burton S (1998). A syndrome of peripheral lipodystrophy, hyperlipidaemia and insulin resistance in patients receiving HIV protease inhibitors. AIDS.

[B3] Carr A, Samaras K, Thorisdottir A, Kaufmann GR, Chisholm DJ, Cooper DA (1999). Diagnosis, prediction, and natural course of HIV-1 protease-inhibitor-associated lipodystrophy, hyperlipidaemia, and diabetes mellitus: a cohort study. Lancet.

[B4] Thiebaut R, Malvy D, Marimoutou C, Davis F (2000). Anthropometric indices as predictors of survival in AIDS adults. Aquitaine Cohort, France, 1985–1997. Groupe d'Epidemiologie Clinique du Sida en Aquitaine (GECSA). Eur J Epidemiol.

[B5] Walli R, Herfort O, Michl GM (1998). Treatment with protease inhibitors associated with peripheral insulin resistance and impaired oral glucose tolerance in HIV-1-infected patients. AIDS.

[B6] Dube MP, Johnson DL, Currier JS, Leedom JM (1997). Protease inhibitor-associated hyperglycaemia. Lancet.

[B7] Behrens GM, Stoll M, Schmidt RE (2000). Lipodystrophy syndrome in HIV infection: what is it, what causes it and how can it be managed?. Drug Saf.

[B8] Fantoni M, Del Borgo C, Autore C, Barbaro G (2002). Metabolic disorders and cardiovascular risk in HIV-infected patients treated with antiretroviral agents. Ital Heart J.

[B9] John M, Nolan D, Mallal S (2001). Antiretroviral therapy and the lipodystrophy syndrome. Antivir Ther.

[B10] Vigouroux C, Gharakhanian S, Salhi Y (1999). Diabetes, insulin resistance and dyslipidaemia in lipodystrophic HIV-infected patients on highly active antiretroviral therapy (HAART). Diabetes Metab.

[B11] Purnell JQ, Zambon A, Knopp RH (2000). Effect of ritonavir on lipids and post-heparin lipase activities in normal subjects. AIDS.

[B12] Hogg RS, O'Shaughnessy MV, Gataric N (1997). Decline in deaths from AIDS due to new antiretrovirals. Lancet.

[B13] Hogg RS, Yip B, Kully C (1999). Improved survival among HIV-infected patients after initiation of triple-drug antiretroviral regimens. CMAJ.

[B14] Allain CC, Poon LS, Chan CS, Richmond W, Fu PC (1974). Enzymatic determination of total serum cholesterol. Clin Chem.

[B15] Naito HK, David JA (1984). Laboratory considerations: determination of cholesterol, triglyceride, phospholipid, and other lipids in blood and tissues. Lab Res Methods Bio Med.

[B16] Warnick GR, Albers JJ (1978). Heparin – Mn2+ quantitation of high-density-lipoprotein cholesterol: an ultrafiltration procedure for lipemic samples. Clin Chem.

[B17] Friedewald WT, Levy RI, Fredrickson DS (1972). Estimation of the concentration of low-density lipoprotein cholesterol in plasma, without use of the preparative ultracentrifuge. Clin Chem.

[B18] Gonzales G, Estrada M, Ferrer C, Astarioa I, Lahera I (1990). Use of serum cholesterol/triglyceride ratio to discern for which individuals the Friedewald formula can be used confidently. Clin Chem.

[B19] Liang K, Zeger S (1993). Regression analysis for correlated data. Ann Rev Public Health.

[B20] Wood E, Hogg RS, Yip B, Harrigan PR, O'Shaughnessy MV, Montaner JS (2004). The impact of adherence on CD4 cell count responses among HIV-infected patients. J Acquir Immune Defic Syndr.

[B21] Wood E, Hogg RS, Yip B, Harrigan PR, O'Shaughnessy MV, Montaner JS (2003). Effect of medication adherence on survival of HIV-infected adults who start highly active antiretroviral therapy when the CD4+ cell count is 0.200 to 0.350 × 10(9) cells/L. Ann Int Med.

[B22] Rhew DC, Bernal M, Aguilar D, Iloeje U, Goetz MB (2003). Association between protease inhibitor use and increased cardiovascular risk in patients infected with human immunodeficiency virus: a systematic review. Clin Infec Dis.

[B23] Martinez E, Arnaiz JA, Podzamczer D (2003). Substitution of nevirapine, efavirenz, or abacavir for protease inhibitors in patients with human immunodeficiency virus infection. N Engl J Med.

[B24] Fisac C, Virgili N, Ferrer E (2003). A comparison of the effects of nevirapine and nelfinavir on metabolism and body habitus in antiretroviral-naive human immunodeficiency virus-infected patients: a randomized controlled study. J Clin Endocrinol Metab.

[B25] Isomaa B, Almgren P, Tuomi T (2001). Cardiovascular morbidity and mortality associated with the metabolic syndrome. Diabetes Care.

[B26] (2001). Executive Summary of The Third Report of The National Cholesterol Education Program (NCEP) Expert Panel on Detection, Evaluation, And Treatment of High Blood Cholesterol In Adults (Adult Treatment Panel III). JAMA.

[B27] Austin MA, Hokanson JE, Edwards KL (1998). Hypertriglyceridemia as a cardiovascular risk factor. Am J Card.

[B28] Hokanson JE, Austin MA (1996). Plasma triglyceride level is a risk factor for cardiovascular disease independent of high-density lipoprotein cholesterol level: a meta-analysis of population-based prospective studies. J Cardiovasc Risk.

[B29] Bozzette SA, Ake CF, Tam HK, Chang SW, Louis TA (2003). Cardiovascular and cerebrovascular events in patients treated for human immunodeficiency virus infection. N Engl J Med.

[B30] Friis-Moller N, Sabin CA, Weber R (2003). Combination antiretroviral therapy and the risk of myocardial infarction. N Engl J Med.

[B31] Eberly LE, Stamler J, Neaton JD (2003). Relation of triglyceride levels, fasting and nonfasting, to fatal and nonfatal coronary heart disease. Arch Int Med.

[B32] Robins JM, Hernan MA, Brumback B (2000). Marginal structural models and causal inference in epidemiology. Epidemiology.

[B33] Turner D, Schapiro JM, Brenner BG, Wainberg MA (2004). The influence of protease inhibitor resistance profiles on selection of HIV therapy in treatment-naive patients. Antivir Ther.

[B34] Squires K, Lazzarin A, Gatell JM (2004). Comparison of Once-Daily Atazanavir With Efavirenz, Each in Combination With Fixed-Dose Zidovudine and Lamivudine, As Initial Therapy for Patients Infected With HIV. J Acquir Immune Defic Syndr.

[B35] Wood R, Phanuphak P, Cahn P (2004). Long-Term Efficacy and Safety of Atazanavir With Stavudine and Lamivudine in Patients Previously Treated With Nelfinavir or Atazanavir. J Acquir Immune Defic Syndr.

[B36] Haas DW, Zala C, Schrader S (2003). Therapy with atazanavir plus saquinavir in patients failing highly active antiretroviral therapy: a randomized comparative pilot trial. AIDS.

[B37] Sanne I, Piliero P, Squires K, Thiry A, Schnittman S (2003). Results of a phase 2 clinical trial at 48 weeks (AI424-007): a dose-ranging, safety, and efficacy comparative trial of atazanavir at three doses in combination with didanosine and stavudine in antiretroviral-naive subjects. J Acquir Immune Defic Syndr.

[B38] Murphy RL, Sanne I, Cahn P (2003). Dose-ranging, randomized, clinical trial of atazanavir with lamivudine and stavudine in antiretroviral-naive subjects: 48-week results. AIDS.

[B39] Palella FJ, Delaney KM, Moorman AC (1998). Declining morbidity and mortality among patients with advanced human immunodeficiency virus infection. HIV Outpatient Study Investigators. N Engl J Med.

[B40] Clotet B, Negredo E (2003). HIV protease inhibitors and dyslipidemia. AIDS Rev.

[B41] Penzak SR, Chuck SK (2002). Management of protease inhibitor-associated hyperlipidemia. Am J Cardiovas Drugs.

[B42] Grinspoon S, Carr A (2005). Cardiovascular risk and body-fat abnormalities in HIV-infected adults. N Engl J Med.

[B43] Nissen SE, Tuzcu EM, Schoenhagen P (2004). Effect of intensive compared with moderate lipid-lowering therapy on progression of coronary atherosclerosis: a randomized controlled trial. JAMA.

[B44] Cannon CP, Braunwald E, McCabe CH (2004). Intensive versus moderate lipid lowering with statins after acute coronary syndromes. N Engl J Med.

[B45] Henry K, Melroe H, Huebesch J, Hermundson J, Simpson J (1998). Atorvastatin and gemfibrozil for protease-inhibitor-related lipid abnormalities. Lancet.

[B46] Bloomfield RH, Davenport J, Babikian V (2001). Reduction in stroke with gemfibrozil in men with coronary heart disease and low HDL cholesterol: The Veterans Affairs HDL Intervention Trial (VA-HIT). Circulation.

[B47] Robins SJ, Collins D, Wittes JT (2001). Relation of gemfibrozil treatment and lipid levels with major coronary events: VA-HIT: a randomized controlled trial. JAMA.

